# Design of Topical Moxifloxacin Mucoadhesive Nanoemulsion for the Management of Ocular Bacterial Infections

**DOI:** 10.3390/pharmaceutics14061246

**Published:** 2022-06-12

**Authors:** Ahmed Adel Ali Youssef, Ruchi Thakkar, Samir Senapati, Poorva H. Joshi, Narendar Dudhipala, Soumyajit Majumdar

**Affiliations:** 1 Department of Pharmaceutics and Drug Delivery, School of Pharmacy, University of Mississippi, Oxford, MS 38677, USA; aayousse@go.olemiss.edu (A.A.A.Y.); rthakkar@go.olemiss.edu (R.T.); ssenapat@go.olemiss.edu (S.S.); phjoshi@go.olemiss.edu (P.H.J.); ndudhipa@olemiss.edu (N.D.); 2 Department of Pharmaceutical Technology, Faculty of Pharmacy, Kafrelsheikh University, Kafrelsheikh 33516, Egypt; 3 Research Institute of Pharmaceutical Sciences, University of Mississippi, Oxford, MS 38677, USA

**Keywords:** ocular bacterial infections, moxifloxacin, nanoemulsion, transcorneal, mucoadhesive agent, ex vivo

## Abstract

Ocular bacterial infections can lead to serious visual disability without proper treatment. Moxifloxacin (MOX) has been approved by the US Food and Drug Administration as a monotherapy for ocular bacterial infections and is available commercially as an ophthalmic solution (0.5% *w*/*v*). However, precorneal retention, drainage, and low bioavailability remain the foremost challenges associated with current commercial eyedrops. With this study, we aimed to design a MOX-loaded nanoemulsion (NE; MOX-NE) with mucoadhesive agents (MOX-NEM) to sustain MOX release, as well as to overcome the potential drawbacks of the current commercial ophthalmic formulation. MOX-NE and MOX-NEM formulations were prepared by hot homogenization coupled with probe sonication technique and subsequently characterized. The lead formulations were further evaluated for in vitro release, ex vivo transcorneal permeation, sterilization, and antimicrobial efficacy studies. Commercial MOX ophthalmic solution was used as a control. The lead formulations showed the desired physicochemical properties and viscosity. All lead formulations showed sustained release profiles a period of more than 12 h. Filtered and autoclaved lead formulations were stable for one month (the last time point tested) under refrigeration and at room temperature. Ex vivo transcorneal permeation studies revealed a 2.1-fold improvement in MOX permeation of the lead MOX-NE formulation compared with Vigamox^®^ eyedrops. However, MOX-NEM formulations showed similar flux and permeability coefficients to those of Vigamox^®^ eyedrops. The lead formulations showed similar in vitro antibacterial activity as the commercial eyedrops and crude drug solution. Therefore, MOX-NE and MOX-NEM formulations could serve as effective delivery vehicles for MOX and could improve treatment outcomes in different ocular bacterial infections.

## 1. Introduction

Ocular bacterial infections present in many disease categories, including keratitis, conjunctivitis, blepharitis, and endophthalmitis [1,2]. Bacterial keratitis (BK) is the inflammation of the cornea due to bacteria, which should be considered an ophthalmic emergency. Bacterial invasion of the cornea can interfere with the pathway of light entering the eye globe and destroy the intact epithelial cells covering the cornea, which could result in irreversible visual impairment if left untreated [3]. The most predominant Gram-positive bacteria isolated in BK are *Staphylococcus aureus*, *Streptococcus pneumoniae*, *Streptococcus viridans*, and *Enterococcus* spp., whereas the most predominant Gram-negative bacteria include *Moraxella lacunata*, *Pseudomonas aeruginosa*, *Haemophilus influenzae*, *Haemophilus parainfluenzae*, *Serratia marcescens*, *Microbacterium lacunata*, and *Microbacterium liquefaciens* [1,4]. The true burden of BK is not known; however, estimates in the US range from 25,000 to 71,000 cases each year, and worldwide rates may exceed 2.0–3.5 million cases annually [5,6].

Bacterial conjunctivitis (BC, i.e., pink eye) is the inflammation of the mucosal lining of the eyelids, which extends to the white sclera and peripheral cornea due to bacteria. BC can spread to involve the whole cornea and results in keratoconjunctivitis. BC is the most commonly diagnosed ocular infection by primary care providers worldwide [6]. The most common isolated bacterial pathogens in BC are *Streptococcus pneumoniae*, *Staphylococcus aureus*, and *Haemophilus influenzae* [2]. In 2005, the BC annual incidence rate was 135 per 10,000 (approximately 4 million cases) in the USA, with an estimated total direct and indirect cost for the treatment of $589 million [7].

Bacterial blepharitis (BB) is the infection of the eyelid margin with bacteria. This infection could also extend to involve the conjunctiva and results in blepharoconjunctivitis. Despite the fact that coagulase-negative staphylococci are the most common bacterium recovered from blepharitis isolates, *Staphylococcus aureus* is the most common cause of bacterial blepharitis [2]. In a 2009 survey, American ophthalmologists and optometrists reported a blepharitis incidence rate of 37% and 47%, respectively [8].

Bacterial endophthalmitis (BE) is a bacterial infection inside the eye globe that involves the vitreous and/or aqueous humor [9]. Coagulase-negative staphylococcus, a normal flora of the ocular adnexa and eyelids, is the most common cause of BE [1]. Outpatient treatment of endophthalmitis has been reported to result in savings in reimbursements of $1.5 to $7.8 million per year in the USA [9].

Topical ocular antibiotic dosage forms can provide a high concentration of antibacterial agents to the cornea and conjunctiva. Therefore, the resistant ocular bacterial isolates identified by the National Committee for Clinical Laboratory Standards could be more susceptible to topical application than to systemic antibiotic treatment. Moxifloxacin (MOX) and gatifloxacin eyedrops were introduced in the ophthalmic market as two novel fluoroquinolones in 2003 [2]. Initially, both antibiotics were approved for BC treatment. Subsequently, their use was extended for BK treatment. These two antibiotics were reported to provide improved coverage against ocular methicillin-resistant *Staphylococcus aureus* (MRSA) [10,11]. Many investigations have reported the safety and efficacy of MOX eyedrops for BB treatment [12,13]. Moreover, intracameral MOX showed better therapeutic outcomes compared to other alternative intracameral antibacterial agents for the prophylaxis and treatment of BE, with minimal risk of ocular toxicity [14,15].

MOX has been approved by the US Food and Drug Administration (USFDA) as a 0.5% *w*/*v* (Vigamox^®^) ophthalmic solution eyedrop for topical ocular applications. However, precorneal elimination of the therapeutic agent due to nasolacrimal drainage and high tear fluid turnover is the major drawback of topical ocular application [16]. Only 1–5% of the therapeutic agent applied to the ocular surface penetrates within the intraocular tissues, whereas the remaining amount overflows from the conjunctival sac and/or is lost through nasolacrimal drainage [16,17]. The low ocular bioavailability necessitates frequent administration (one drop 3 times daily for 7 days), raising the risk for well-reported non-ocular adverse events, such as increased cough, otitis media, pharyngitis, and rhinitis. Effective early treatment of ocular bacterial infections can prevent progression to corneal perforation, endophthalmitis, or blindness [18,19].

Nanoemulsion (NE) formulations are nanosized, thermodynamically stable, isotropic systems with a droplet size in the range of 20–200 nm [20]. NE formulations are known to improve ocular bioavailability, sustain drug release, and facilitate drug distribution to the deeper ocular tissues [16,21]. In addition, NEs have high drug-solubilizing capacity, biocompatibility, and physicochemical stability [21,22]. Moreover, the low surface tension of NEs allows for excellent spreading on the corneal surface and, consequently, better mixing with precorneal tear film, prolonging the contact time between the drug and the ocular surface and improving ocular bioavailability [23]. Furthermore, the sterilization process of NEs is easy and inexpensive, similar to conventional solutions, due to their submicron size [24].

Mucoadhesive polymers are known to prolong the residence time on the ocular surface after topical administration by adhering to the mucin layer and enabling a uniform distribution across the surface of the eye globe [25]. Hence, the incorporation of a mucoadhesive polymer in the NE formulation could result in improved ocular bioavailability and prolonged antibacterial activity [26]. Hypromellose (HPC; hydroxyl propyl methyl cellulose, 0.1–0.6% *w*/*w*) polymers, such as hypromellose 2906 (4000 mPa·s), hypromellose 2910 (4000 mPa·s), hypromellose 2910 (3.0 mPa·s), or hypromellose 2910 (5.0 mPa·s), are widely used in many FDA-approved ophthalmic formulations as a mucoadhesive agent due to their aqueous solubility, biocompatibility, transparency, and rheological properties [25,27]. Povidone (PVP; polyvinylpyrrolidone) is also a recognized polymer for ocular applications and is widely used in many FDA-approved ophthalmic products at concentrations ranging from 0.3 to 15% *w*/*v* [28]. PVP is an inert, non-toxic, biocompatible, biodegradable, mucoadhesive polymer [28].

The objective of the current investigation was to overcome the potential drawbacks of commercial MOX eyedrops by developing MOX-NEs by incorporating mucoadhesive agents (MOX-NEM) that could enhance ocular surface retention and penetration into ocular tissues and reduce the frequency of administration. Accordingly, MOX-NEs were prepared, optimized, and evaluated based on physicochemical characteristics and stability. The lead MOX-NE formulation was then converted into a mucoadhesive NE with the addition of HPMC and PVP polymers as thickening and mucoadhesive agents, respectively. MOX-NE and MOX-NEM formulations were evaluated for in vitro release, ex vivo permeation, and antimicrobial efficacy and compared against commercial MOX ophthalmic solution eyedrops (Vigamox^®^, 0.5% as a base).

## 2. Materials and Methods

### 2.1. Materials

#### 2.1.1. Chemicals and Glassware

MOX was purchased from Fischer Scientific (Hanover Park, IL, USA). MOX-HCL was purchased from Combi-Blocks, Inc. (San Diego, CA, USA). Oleic acid, Tween^®^ 80, and glycerin were purchased from Spectrum Pharmaceuticals (Henderson, NV, USA). Slide-A-Lyzer™ MINI dialysis devices (10 K molecular weight cutoff) were obtained from Fischer Scientific (Hampton, NH, USA). Hydroxypropyl methylcellulose (HPMC K4M) was received as a gift from Colorcon^®^ (Colorcon, Inc., West Point, PA, USA). Polyvinylpyrrolidone (PVP, Plasdone™ K29/32) was purchased from Ashland Global (Wilmington, DE, USA). Solvents used for analysis were of high-performance liquid chromatography (HPLC) grade and were purchased from Fischer Scientific (Hampton, NH, USA). All membrane filters were purchased from MilliporeSigma (St. Louis, MO, USA). Centrifuge tubes, HPLC vials, and scintillation glass vials were acquired from Fischer Scientific (Hampton, NH, USA). Cation-adjusted Mueller Hinton Broth 2 was purchased from MilliporeSigma (St. Louis, MO, USA).

#### 2.1.2. Biological Tissues and Samples

The whole eyes of mixed-gender albino New Zealand rabbits (weight, 4.75–5.75 lbs and; age, 8–12 weeks) were purchased from Pel-Freez Biologicals (Rogers, AR, USA). Microbial strains were obtained from the American Type Culture Collection (ATCC, Manassas, VA, USA).

### 2.2. Methods

#### 2.2.1. HPLC

MOX concentration in all samples was quantified using HPLC, Alliance Waters e2695 separations module, and a Waters 2489 UV/Vis dual absorbance detector. A detection wavelength (λ_max_) of 254 nm was set. The mobile phase consisted of a mixture of phosphate buffer (18 mM) containing 0.1% *v*/*v* triethylamine (pH 2.8, adjusted with dilute phosphoric acid) and methanol (60:40 *v*/*v*) at a flow rate of 1.0 mL/min [29]. Chromatographic separation was achieved within 10 min using a Phenomenex Luna^®^ C_18_ column (250 × 4.6 mm, 5 μ) as a stationary phase with a retention time of 7.1 ± 0.2 min. The samples were analyzed through a Waters chromatography data system and Empower software. The HPLC method was found to be linear over the MOX base concentration range of 1.0–100 μg/mL, with a limit of detection (LOD) and limit of quantitation (LOQ) of 0.8 and 2.4 µg/mL, respectively.

#### 2.2.2. Screening of Oils

The solubility of MOX in various oils was determined by individually adding 10 mg of MOX to 200 mg of the oils in glass vials (3 mL). Then, the drug–oil mixture was vortexed (Vortex Genie^®^ 2, Scientific Industries, Inc., SI™, Bohemia, NY, USA) for 2 min. Next, the MOX–oil mixtures were heated at 80.0 ± 2.0 °C and kept under continuous magnetic stirring (2000 rpm) for 10 min. The mixtures were then allowed to cool to room temperature and examined visually for MOX precipitation. The oil that did not show any precipitation was chosen for formulation preparation.

#### 2.2.3. Preparation of MOX-NE Formulations

Oil-in-water (O/W)-type NEs were prepared by hot homogenization followed by the probe sonication method [16]. The oily phase was prepared by dissolving an accurately weighed amount of MOX base within the selected oil by heating at 80.0 ± 2.0 °C to obtain a clear drug solution. An aqueous phase, prepared by adding Tween^®^ 80 and glycerin to Milli-Q water, was also heated at 80.0 ± 2.0 °C. Then, the hot aqueous phase was added to the heated oil phase dropwise under continuous magnetic stirring at 2000 rpm for 10 min to form a macroemulsion. This macroemulsion was then homogenized using a T25 digital Ultra-Turrax (IKA Works, Inc., Wilmington, NC, USA) at 11,000 rpm for 5 min at 65.0 ± 2.0 °C [16]. The macroemulsion was allowed to cool to room temperature before being subjected to probe sonication at 40% amplitude for 10 min with a 3 mm stepped microtip with a 500 watt power supply at 115 volts (Pulse on; 10 s and Pulse off; 15 s) using a Sonics Vibra-Cell™ sonicator (Newtown, CT, USA) to form NE [30].

#### 2.2.4. Preparation of MOX-NEM Formulations

The NE preparation method, as described above, was followed for the preparation of the NEM formulations. However, the volume of Milli-Q water used to prepare NE was split into two equal parts: one part was used to prepare the aqueous solution of the mucoadhesive agent (HPMC K4M or PVP K29/32), and the other half was used to prepare the aqueous solution of surfactant and glycerin, as described above.

#### 2.2.5. Control Formulation

##### Vigamox^®^ (Alcon Laboratories, Inc., Fort Worth, TX, USA)

A commercial ophthalmic solution of moxifloxacin hydrochloride (0.5% *w*/*v*) was used as a control formulation for the in vitro release, ex vivo permeation, and antibacterial efficacy studies.

##### Crude Drug Solution (MOX-HCL-S)

MOX hydrochloride powder (moxifloxacin hydrochloride 5.45 mg equivalent to 5.0 mg moxifloxacin base) was dissolved in 1 mL saline water to ensure that the concentrations of MOX base in the control and test formulations were equal for antimicrobial efficacy studies.

#### 2.2.6. Measurement of Droplet Size (DS), Polydispersity Index (PDI), and Zeta Potential (ZP)

The MOX-NE and MOX-NEM formulations were evaluated for their DS, PDI, and ZP using a Zetasizer instrument (Nano ZS Zen3600, Malvern Panalytical Inc., Westborough, MA, USA) at 25 °C in disposable, folded, clear, solvent-resistant micro cuvettes (ZEN0040) [31]. The cuvette was rinsed with 0.22 µm filtered Milli-Q water to ensure the absence of dust/particulates in each cuvette before performing the measurement. Prior to measurement, formulations were diluted (100 times) with Milli-Q water. The same diluted formulations were transferred to Zetasizer disposable folded capillary DTS1070 cells for ZP measurement after DS and PDI measurement. All DS, PDI, and ZP measurements were carried out in triplicate.

#### 2.2.7. Drug Content

For drug content analysis, an accurate volume (50 µL) of each NE formulation was added to 950 µL methanol (extracting solvent). This extract was centrifuged (AccuSpin 17R centrifuge, Fisher Scientific, Hanover, IL, USA) at 13,000 rpm for 20 min. Then, the supernatant was diluted 10 times with the mobile phase and analyzed for MOX content using the HPLC method described above.

#### 2.2.8. pH Measurement

pH was measured using a Mettler Toledo pH meter (FiveEasy™, Columbus, OH, USA) equipped with an Inlab^®^ Micro Pro-ISM probe. Before measurement, the pH meter was calibrated using different standard buffers with known pH values of 4.01, 7.00, and 10.01. The pH of each prepared NE or NEM formulation was measured in triplicate.

#### 2.2.9. Viscosity Measurement

A Brookfield cone and plate viscometer (LV-DV-II+ Pro Viscometer, Middleborough, MA, USA) was used to measure the viscosity (η) of all MOX-NE and MOX-NEM formulations using a CPE 52 spindle operated at 10 rpm. Each formulation (1.0 mL) was placed in the viscometer cup plate. The temperature of the cup was maintained at 25 °C using a circulating water bath. Data analysis was performed using Rheocalc software (version 3.3, build 49-1). The viscosity measurements of all formulations were carried out in triplicate.

#### 2.2.10. Stability Studies

The physicochemical stability of NE and NEM formulations was evaluated under refrigerated (RF, 4.0 ± 2.0 °C), room-temperature (RT, 25.0 ± 2.0 °C), and accelerated (40.0 ± 2.0 °C, 75% RH, NE only) storage conditions. MOX-NE and MOX-NEM formulations were evaluated for any change in DS, PDI, ZP, pH, and MOX content under the aforementioned conditions for three months.

#### 2.2.11. Sterilization Process and Stability Assessment

Filtration and autoclaving methods were used for the sterilization of lead NE and NEM formulations. Sterilization of the MOX-NE and MOX-NEM formulations by filtration technique was investigated through 0.22 µm filter pore size. Samples (1.0 mL) were passed through various filter membranes using a 13 mm stainless-steel Swinny filter holder (MilliporeSigma, St. Louis, MO, USA). Different filter membranes were tested, such as Millex^®^ syringe filters with nylon membranes (0.20 µm), Durapore™ (PVDF; polyvinylidene difluoride, 0.20 µm), Fluoropore™ (PTFE; polytetrafluoroethylene, 0.22 µm), and Millipore Express^®^ PLUS (PES; polyethersulfone, 0.22 µm). The filtrate was collected in a glass vial, and the effect of the filtration technique on the physicochemical characteristics of the MOX-NE and MOX-NEM formulations was evaluated.

MOX-NE and MOX-NEM formulations were also subjected to autoclaving (121 °C under 15 psi for 15 min, 3850ELP-B/L-D Tuttnauer autoclave, Heidolph, Germany) in glass vials affixed with indicator tapes for the sterilization process. The sterilization cycle was confirmed by the color change of the indicator tapes attached to the glass vials. After the moist heat sterilization process, formulations were stored at RF and RT for one month (last time point tested) in the same containers and analyzed in the same way as the pre-autoclaved samples.

#### 2.2.12. Scanning Transmission Electron Microscopy (STEM)

STEM analysis was performed using a JSM-7200FLV scanning electron microscope (JOEL, Peabody, MA, USA) attached to a STEM detector with an accelerating voltage of 30 KV. STEM samples were examined according to a negative staining protocol with a solution of UranyLess. A carbon-plated copper grid was placed above one drop (20 µL) of the MOX-NE formulation for 60 s, and the excess sample was removed with the aid of a filter paper after grid removal from the formulation surface. The grid was then washed by dipping in distilled water, and the excess water was removed from the grid using filter paper. Next, the grid was placed above one drop (20 µL) of the staining solution for 60 s, and the excess stain was also drawn off the grid with the aid of a filter paper. The grid was allowed to air dry for a few minutes. The grid was examined under the scanning transmission electron microscope at 65 K times magnification power.

#### 2.2.13. In Vitro Release Studies

Phosphate-buffered saline (PBS; pH 7.4) containing 5.0% *w*/*v* hydroxypropyl beta-cyclodextrin (HPβCD) was used as the receiver medium for in vitro release and ex vivo transcorneal permeation studies. The media were selected based on our earlier investigations [32]. In vitro release profiles of MOX from Vigamox^®^, MOX-NE, and MOX-NEM formulations were evaluated using the dialysis method. Test and control formulations (200 μL) were added to 0.5 mL cup-like dialysis devices (donor compartment) and fitted on the top of scintillation glass vials (receiver compartment). The content of the receiver compartment was maintained under continuous magnetic stirring at 34.0 ± 2.0 °C on the top of a multi-stationed magnetic stirrer (IKA Works, Inc., Wilmington, NC, USA) at 500 rpm. Samples (1 mL) were collected from the receiver compartment and replaced with an equivalent volume of a freshly prepared release medium at scheduled time points (0.5, 1, 2, 3, 4, 6, 8, and 12 h). Samples were analyzed after collection using the HPLC method mentioned above. The time for 50% release of the loaded drug (T_50%_) was calculated. The release data were fitted to different mathematical release models using DDSolver software, a free add-on program for Microsoft Excel (Office365, 2016, Redmond, WA, USA), to elucidate the possible release mechanism.

#### 2.2.14. Transcorneal Permeation Studies

The ex vivo transcorneal permeation of MOX from the test and control formulations was evaluated on corneas isolated from whole rabbit eyes that were shipped overnight over ice in Hanks’ balanced salt solution. Ex vivo permeation testing was performed using a vertical Franz diffusion apparatus (PermeGear^®^ Inc., Hellertown, PA, USA). The corneas were excised carefully upon arrival and washed with IPBS solution (pH 7.4). Each excised cornea was crimped between the two chambers of each vertical Franz diffusion cell (spherical joint), with the corneal epithelium facing the donor chamber that contained the formulation. The receiver chamber contained a solution of HPβCD in PBS (5.0 mL, 5.0% *w*/*v*, pH 7.4) kept under continuous magnetic stirring at 34.0 ± 2.0 °C. Aliquots (0.5 mL) were withdrawn from the receiver compartment at scheduled time points and replenished with an equal volume of the receiver medium. The aliquots were quantified for MOX using the HPLC method described above. The cumulative amount of MOX-permeated (*Q_n_*), steady-state flux (*J_ss_*) and permeability coefficient (*P_eff_*) across the excised rabbit corneas were calculated.

*Q_n_* was calculated based on the following formula:

$$Q_{n}{=V}_{r}C_{r(n)}+\overset{x=n}{\underset{x=1}{\sum }}V_{s(x-1)}C_{r(x-1)}$$

where *n* is the sampling time point, *V_r_* is the volume in the receiver compartment (mL), *V_S_* is the volume of the aliquot collected at the nth time point (mL), and *C_r(n)_* is the drug concentration in the receiver compartment at the nth time point (µg/mL).

*J_ss_* was calculated by the following formula:

*J_ss_* = (*dQ*/*dt*)/*A*

where (*dQ*/*dt*) is the rate of transcorneal permeation calculated using the slope of *Q_n_* versus the time plot, and *A* is the effective area of transcorneal permeation (0.64 cm^2^).

The transcorneal permeability coefficient was calculated using the following formula:

*P_eff_* = *J_ss_*/*C*_0_

where *C*_0_ is the initial donor concentration for MOX.

#### 2.2.15. Antimicrobial Efficacy

The antimicrobial activity of MOX-NE and MOX-NEM formulations was evaluated against methicillin-resistant *Staphylococcus aureus* ATCC 1708 (MRSA). Antimicrobial susceptibility testing was performed following a modified version of the Clinical and Laboratory Standards Institute (CLSI, 2012) methods by employing the most widely used susceptibility testing method, broth microdilution. MOX-NE and MOX-NEM formulations were serially diluted using cation-adjusted Mueller Hinton Broth 2 assay medium (pH 7.0). Then, the diluted samples (100 µL) were transferred to small, disposable, plastic “microdilution” trays (96 well). Inocula were prepared by correcting the OD630 of the bacterial suspensions in the incubation broth to provide recommended inocula as per CLSI protocol. Next, 5.0% Alamar Blue™ was added to MRSA microdilution trays. Crude MOX-HCL solution was used as a positive control in each assay. The optical density was measured for each of the panel wells by a Bio-Tek plate reader before and after incubation at 35 °C for 24 h. The minimum inhibitory concentration (MIC) was determined for all tested formulations and was defined as the lowest test MOX concentration that results in no visual growth. All experiments were carried out in triplicate.

#### 2.2.16. Statistical Analysis

All results are presented as mean ± standard deviation. SPSS 28 software (IBM^®^, Armonk, NY, USA) was used for statistical analysis of data. The significant difference between the data was compared at a *p*-value less than 0.05 (*p* < 0.05).

## 3. Results and Discussion

### 3.1. Screening of Oils

The solubility of the drug in the oily phase, which constitutes the dispersed phase in the O/W emulsion, is the most important criterion during the development of NE formulations for hydrophobic drugs. Drug loading in the NE is also dependent on the drug solubility within the surfactant micelles used to prepare NE formulations; however, drug precipitation could occur upon contact with biological fluids because the dilution of the NE formulation could affect the solubilizing power of the surfactant [16,33]. Therefore, the selection of oil is a critical step during the design of NE formulations. The solubility of MOX in various oils was evaluated based on visual examination (Table 1). MOX dissolved in oleic acid did not show any precipitation after cooling the drug–oil mixture to RT. Therefore, oleic acid was selected to prepare the NE and NEM formulations. Oleic acid is a long-chain unsaturated fatty acid that is widely used for ocular administration due to its biocompatibility and well-tolerated safety profile [34]. In addition, oleic acid has often been used in ophthalmic formulations, as it acts as a powerful penetration enhancer for both lipophilic and hydrophilic drugs, even at a concentration of 0.05% *v*/*v*, as it increases the fluidity of intercellular lipid barriers [35].

### 3.2. Preparation of MOX-NE Formulations

The compositions of the different NE formulations tested are presented in Table 2. MOX-NE formulations were prepared using oleic acid as the oily phase, selected based on the solubility of MOX in different oils. Tween^®^ 80 and glycerin represented the aqueous phase. According to the FDA inactive ingredient database, concentrations of Tween^®^ 80 and glycerin (tonicity-adjusting agent) of as much as 4.0 and 2.25% *w*/*v*, respectively, are present in FDA-approved topical ocular applications. Nonionic hydrophilic surfactants, such as Tween^®^ 80 with HLB > 10, have been reported for the preparation of uniform stable O/W NEs [36]. Drug loading was maintained at 0.5% *w*/*v* in the MOX-NE formulations to ensure that the concentrations of MOX base in the commercial (control) and test NE formulations were equal.

The MOX-NE formulations were optimized based on different oil concentrations (1, 2.5, and 5.0% *w*/*v*) and surfactant concentrations (0.75, 2.0, 3.0, and 4.0% *w*/*v*). The formulations were subjected to a one-month follow-up study at 4 °C based on visual examination. MOX-NE formulations (F1-F8) showed drug precipitations within 17 days after preparation. This could be due to an insufficient amount of oil to dissolve the drug. Formulation F9 showed phase separation, probably due to an insufficient amount of surfactant to stabilize the emulsion [37]. However, MOX-NE formulations F10-F12 were observed to be stable and did not show any precipitation or cracking, suggesting a sufficient amount of oil and surfactant. Therefore, formulations F10-F12 were selected for further evaluation and modifications.

### 3.3. Physicochemical Characteristics of MOX-NE Formulations

The effect of Tween^®^ 80 concentration on the formulation was investigated. Results of varying Tween^®^ 80 concentrations in the MOX-NE formulations are presented in Table 3. Increasing Tween^®^ 80 concentrations from 0.75% to 4.0% *w*/*v* decreased the DS significantly (*p* < 0.05) from 174.1 ± 5.9 to 124.7 ± 1.5 nm. No significant change was observed in PDI, ZP, viscosity, and drug content. The significant effect of increasing Tween^®^ 80 concentration on DS could be attributed to the significant reduction in surface tension and surface free energy that was generated during the homogenization and ultrasonication steps [38,39].

The DS of the nanocarriers is important for the adhesion and interaction with the ocular epithelial cells. Smaller particles/globules (100–200 nm) can be transported by receptor-mediated endocytosis uptake, whereas larger particles are internalized by phagocytosis [30,40,41]. PDI values reveal the width of DS distribution, and a low PDI value (<0.3) demonstrates that the nanoparticulate system is a uniform dispersion with narrow DS distribution [42]. ZP within ±30 mV (absolute value) provides good physical stability for NEs, and the stability becomes excellent when ZP values approach ±60 mV. However, a ZP value of >±20 mV provides only short-term NE stability, whereas the −5 to +5 mV range could suggest rapid aggregation of the dispersed oil globules [42]. Therefore, the successful NE formulations (F10–F12) showed good DS (<200 nm), PDI (<0.2), and ZP (>−30 mV) values.

Each NE formulation should have MOX content within the acceptance limits of the label’s content to ensure the consistency of the dosage form units. The drug content of the three successful NE formulations was in the range of 99.1 ± 3.8–100.8 ± 7.3% of the theoretical value.

### 3.4. Addition of Mucoadhesive Agents to the MOX-NE Formulation

In our previous studies with Δ^9^-tetrahydrocannabinol-valine-hemisuccinate NEs, we observed that increasing the Tween^®^ 80 concentration to 2.0% *w*/*v* enabled an autoclaving process [25]. Therefore, the MOX-NE F10 (2.0% *w*/*v* surfactant) formulation was selected for further development with the addition of mucoadhesive agents.

HPMC K_4_M is present in FDA-approved ophthalmic formulations to concentrations up to 0.5% *w*/*v*. HPMC is used as a viscosity enhancer, gelling agent, and mucoadhesive agent in eyedrops or as a hydrophilic polymeric matrix in ocular films and inserts [26]. HPMC K_4_M also exhibits a high swelling capacity, which can further aid in providing sustained drug delivery vehicles [43]. Such vehicles help to effectively transfer the target molecule to the site of action [44]. PVP is widely used to increase the residence time of many topical ophthalmic products by way of its viscosity-enhancing and mucoadhesive properties. It is also used as an effective lubricant for dry eye disease, as well as a suspending agent, wetting agent, and stabilizer in ophthalmic suspensions due to its amphiphilic properties [28]. Moreover, this amphiphilic nature offers good solubility in water and organic solvents [45]. HPMC K_4_M and PVP K29/32 were evaluated at a concentration of 0.4% *w*/*v* (Table 2). The selected polymer concentration imparts viscosity while allowing for easy topical ocular application (≤50 cP) based on our earlier investigations [25]. Incidentally, initial trials with Carbopol^®^ 940 NF revealed that the NE formulations were physically unstable (data not presented).

### 3.5. Physicochemical Characteristics of MOX-NEM Formulations

The physicochemical characteristics of the MOX-NE formulations with and without the addition of the HPMC K4M and PVP to the NE formulations as mucoadhesive agents are illustrated in Table 4. Upon the addition of the PVP and HPMC to the MOX-NEs, there was no significant difference observed in DS, PDI, and ZP. Moreover, the drug content for all the formulations was found to be in the range of 98.3 ± 2.5 to 101.0 ± 1.3%.

### 3.6. pH and Viscosity

The normal human tear pH range is 6.5 to 7.6, with a mean value of 7.0 [46]. The eye can tolerate topically administered products with pH values in the range of 3.0 to 8.6, depending on the buffering capacity of the formulation (USP). We observed that the incorporation of the mucoadhesive agents did not significantly affect the pH of the MOX-NE formulation. All NE formulations had pH values ranging from 5.4 ± 0.1 to 5.5 ± 0.1, which is favorable for ocular application (Table 4).

Although viscosity evaluation is not a compendial test, it is an essential part of the manufacturer’s specification of the ocular product because viscosity can affect product performance. A viscosity of up to 50 cP, allows for easy topical ocular application [47]. The viscosity of MOX-NE and MOX-NEM formulations was measured using a Brookfield cone and plate viscometer (Table 4); we observed that the viscosity of the NE formulation (5.6 ± 0.5 cP) was increased significantly (*p* < 0.05) after the inclusion of HPMC K_4_M (F13; 26.5 ± 0.9 cP) and PVP K29/32 (F14; 27.9 ± 0.3 cP) polymers within the formulation. The increase in viscosity of the NE formulation after the addition of the high-molecular-weight mucoadhesive polymers could be due to the strong internal friction between the randomly coiled and swollen polymer chains and the surrounding water molecules [48]. There was no significant (*p* > 0.05) difference between the viscosity values obtained for the mucoadhesive formulations.

### 3.7. Sterilization by Filtration

All ophthalmic dosage forms must be sterile and comply with the pharmacopeial tests for sterility. Filtration is widely adopted as a sterilization approach for NE formulations due to their small DS, which is less than the maximum nominal pore size (0.22 µm) of membrane filters utilized in this sterilizing process [16]. Thus, filtration of the three lead formulations was tested using different types of filter membranes. We observed that all three formulations could be easily filtered through Durapore™, nylon, and PES membrane filters; however, they faced resistance through the Fluoropore™ membrane filter.

**Figure 1:**
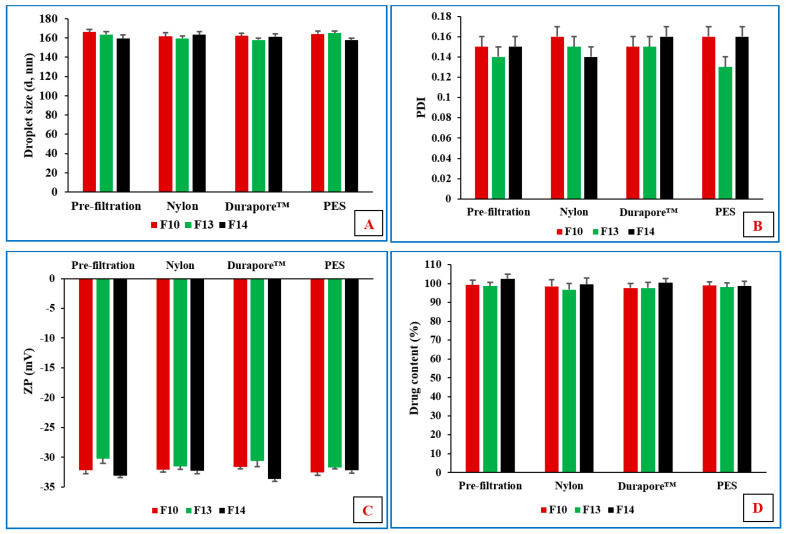
Effect of filtration on (**A**) droplet size (d, nm), (**B**) PDI, (**C**) zeta potential (mV), and (**D**) drug content (%) of F10, F13, and F14 formulations (mean ± SD, n = 3).

Durapore™, nylon, and PES are hydrophilic membrane filters, whereas Fluoropore™ is a hydrophobic membrane filter. This could be the reason that all formulations passed through the hydrophilic filters but faced resistance through the hydrophobic filter materials because water constitutes the major composition of O/W NEs. Filtration could affect the particle size and distribution, along with the drug content, during the sterilization process. Therefore, the physicochemical characterization of all formulations was investigated before and after the filtration process. No significant (*p* > 0.05) difference was observed in terms of DS, PDI, ZP, and MOX content between the pre- and post-filtration formulations (Figure 1).

Steam sterilization is probably the most widely used of all sterilization methods. As opposed to aseptic manufacture, terminal sterilization in the final dosage form container is the preferred approach from a regulatory point of view. Owing to the advantage as a terminal sterilization process, moist-heat sterilization was also investigated for the NE and NEM formulations. F10, F13, and F14 formulations were selected to evaluate moist-heat sterilization process stability based on a three-month stability study of the unsterilized formulations. The pre- and post-moist-heat sterilization physicochemical characteristics are presented in Table 5. The autoclaved formulations remained stable at RF and RT; DS, PDI, ZP, pH, and drug content did not show a significant (*p* < 0.05) change in comparison to the pre-autoclaved formulation for one month. The sterilized formulations maintained high negative ZP values (≥30 mV), reflecting a high negative surface charge for adequate droplet–droplet-repulsive forces, improving NE and NEM stability.

### 3.8. STEM

**Figure 2:**
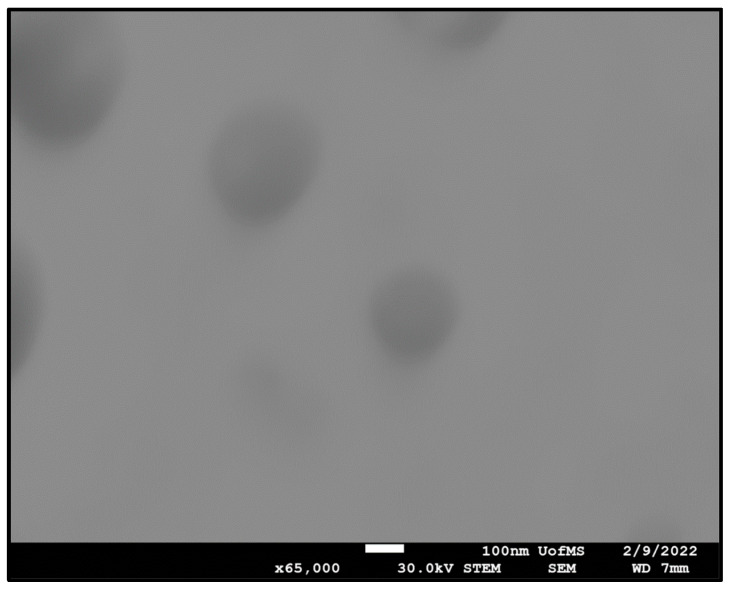
Scanning transmission electron micrograph of F10 formulation at ×65 k magnification.

STEM images obtained for the F10 formulation exhibited spherical oil droplets with a diameter of less than 200 nm, as shown in Figure 2. The DS distribution profile was in close agreement with Zetasizer results.

### 3.9. Antibacterial Activity of MOX-NE and MOX-NEM

*Staphylococci* are the most common causative agents of ocular bacterial infections, and MRSA is associated with persistent and complicated ocular infections [49]. The goal of this study was to evaluate the antimicrobial efficacy of all developed formulations in comparison to commercial Vigamox^®^ eyedrops and the crude MOX solution against MRSA. The MIC 90 value (6.25 µg/mL) obtained for all three formulations was the same as that of Vigamox^®^ eyedrops and crude MOX-HCL solution, as shown in Table 6. This implies that all lead NE formulations were as effective as the commercial formulation in terms of their antimicrobial efficacy against this vision-threatening bacterium.

### 3.10. In Vitro Release Studies

**Figure 3:**
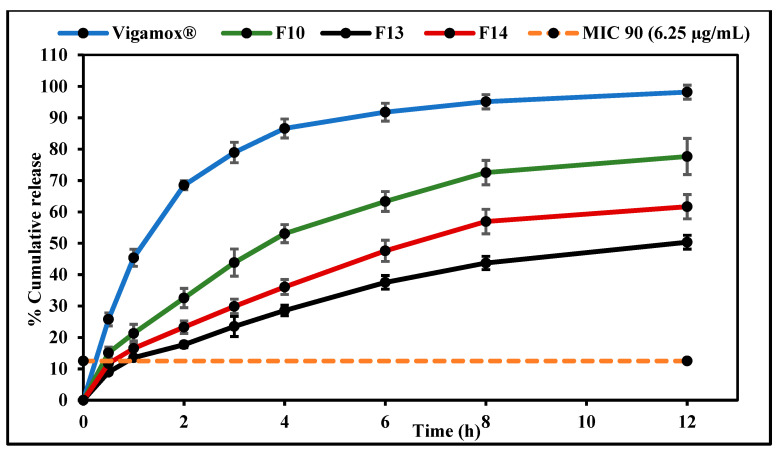
In vitro release profiles of moxifloxacin from Vigamox^®^, F10, F13, and F14 formulations determined by a Thermo Scientific™ Slide-A-Lyzer™ MINI dialysis device (10 K MWCO) (mean ± SD, n = 3).

In vitro release testing of MOX from F10, F13, F14, and Vigamox^®^ formulations was performed by employing the dialysis method; the in vitro drug release profiles are graphically illustrated in Figure 3. The T_50%_ values of the release profiles of the control, F10, F13, and F14 formulations were 1.2, 3.6, 12.0, and 6.4 h, respectively. Thus, F10, F13, and F14 formulations sustained the release of the loaded drug compared to the commercial formulation. However, drug release from the PVP-containing NE formulation (F14) was slightly faster than that from the HPMC-containing formulation (F13). The entrapment of drug molecules within the NE formulation oil globules and slow drug diffusion from the oil phase to the aqueous phase may have contributed to the sustained release behavior from the NE and NEM formulations [48]. Moreover, the retarded drug release rates from F13 and F14 compared to the F10 formulation could be attributed to the increased viscosity imparted by the addition of the mucoadhesive polymers to the formulation [50]. These observations are consistent with many previously reported investigations [51,52].

Although the inclusion of a mucoadhesive agent prolongs the contact time of the formulation with the ocular surface, it could inhibit the diffusion of the drug out of the formulation [25]. However, the release data demonstrate that all test formulations achieved MOX concentrations greater than the obtained MIC 90 (6.25 µg/mL) within the first hour—10.6 µg/mL for F10, 6.8 µg/mL for F13, and 8.2 µg/mL for F14—indicating that the MIC against MRSA was attained across at release time points.

Four conventional release models, including zero-order, first-order, Higuchi, and Korsmeyer–Peppas, were fitted to each of the three data sets, and a regression analysis was performed in each case to verify the goodness of fit. The release model that showed the highest value for the coefficient of determination (R^2^) was regarded as the best model to describe release kinetics, as illustrated in Table 7. Mathematical model fitting revealed that the dissolution profile of all developed formulations followed the Korsmeyer–Peppas model. All calculated slope values (n) of the Korsmeyer–Peppas model (0.5 < n < 1.0) indicated non-Fickian drug release profiles controlled by a diffusion mechanism. The general sustained release behavior of O/W NE is due to the fact that the release of hydrophobic drugs from this NE type involves many consecutive steps, starting from partitioning (diffusion) of the hydrophobic drug from oil into surfactant and then into the aqueous phase [16].

### 3.11. Ex Vivo Transcorneal Permeation

**Figure 4:**
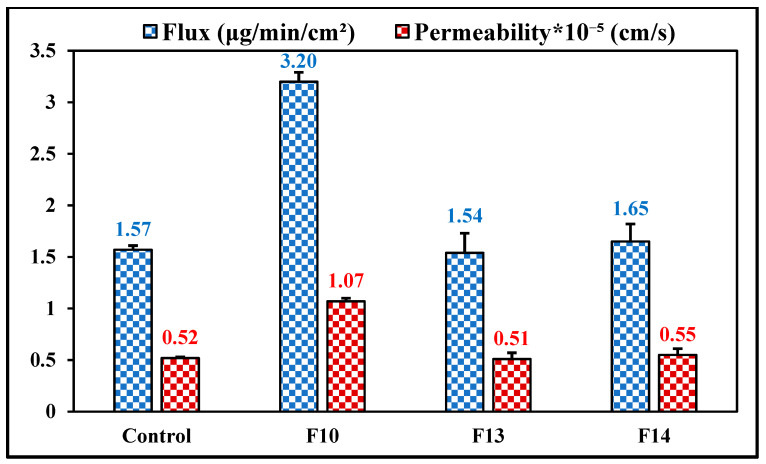
Transcorneal flux and permeability of moxifloxacin from F10, F13 (HPMC K4M), F14 (PVP K29/32), and control (Vigamox^®^) formulations through isolated rabbit corneas (mean ± SD, n = 3).

The rabbit eye is considered a reference animal model for transcorneal permeation studies because of the high similarity between rabbit and human eyes [53]. Flux and permeability of MOX across rabbit corneas from the commercial solution, F10, F13, and F14 formulations were studied, and the data are shown in Figure 4. The flux of MOX from the mucoadhesive NE formulations—1.54 ± 0.19 μg/min/cm^2^ for F13 and 1.65 ± 0.17 μg/min/cm^2^ for F14—did not show a significant difference (*p* > 0.05) when compared to the MOX flux from the control eyedrops (1.57 ± 0.04 μg/min/cm^2^). However, the transcorneal flux of MOX from F10 formulations was approximately 2.0-fold higher compared to the control eyedrops and the mucoadhesive NE formulations. Consequently, the corneal permeation of MOX was approximately 2.1-fold greater from NE as compared to that of the commercial and mucoadhesive NE formulations. The observed improved drug flux from NE, as compared to the mucoadhesive NE formulations, could be due to the fact that the addition of the mucoadhesive polymer could slow the diffusion of the drug out of the formulation to the cornea; however, this phenomenon could be beneficial for prolonging ocular surface retention and sustaining penetration into the intraocular tissues [25].

A possible reasons for the improved corneal penetration of drugs loaded in the NE formulation could be that the presence of surfactants increases the membrane permeability, thereby increasing drug uptake [23]. Moreover, nanoparticles with a particle size of less than 200 nm could be internalized by a receptor-mediated endocytosis uptake mechanism through the corneal tissue [36]. In addition, oleic acid has been reported to improve the ocular drug delivery of both lipophilic and hydrophilic compounds [35]. Until now, no clear mechanisms have been reported for the ocular penetration-enhancing effect of oleic acid; however, oleic acid could produce transient ultrastructure changes in corneal epithelium, offering pathways for actives by perturbation of the highly ordered lipid bilayer.

Some previous studies evaluated MOX-loaded nanoparticle formulations. Shah et al. prepared NE formulations with 12% *w*/*v* Tween^®^ 80, which is in excess of the FDA inactive ingredient database concentration for topical ocular applications by three times, which could lead to irritation and toxicity [54]. Gade et al. prepared MOX (0.2% *w*/*v*)-loaded nanostructured lipid carriers (NLC; MOX-NLC). The low drug loading, which lacks a comparison with commercial eyedrops, makes evaluation of any improvement in permeation difficult [55]. In another study, MOX ocular inserts were prepared by Sebastián et al. [56]. However, these ocular inserts were prepared by the solvent cast approach, which raises concerns about residual solvents within the final dosage form, and the prepared inserts were non-biodegradable, therefore requiring removal after each use, which could reduce patient compliance.

### 3.12. Stability Studies of NE and Mucoadhesive NE Formulations

**Figure 5:**
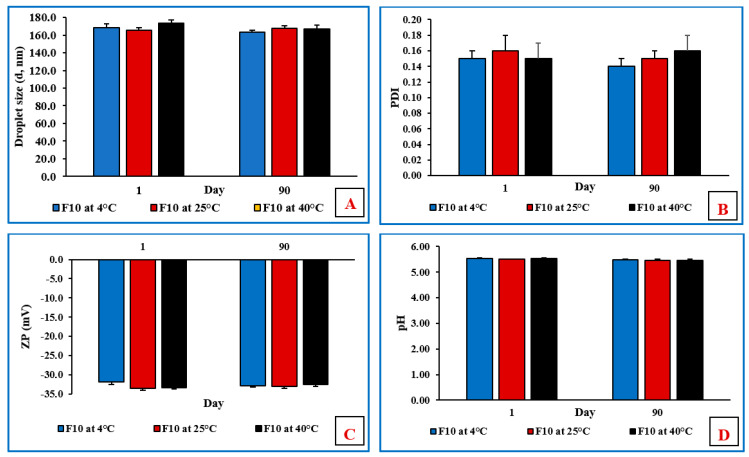
(**A**) Droplet size, (**B**) polydispersity index, (**C**) zeta potential, and (**D**) pH of F10 formulation over three months of storage at 4 °C, 25 °C, and 40 °C (mean ± SD, n = 3).

**Figure 6:**
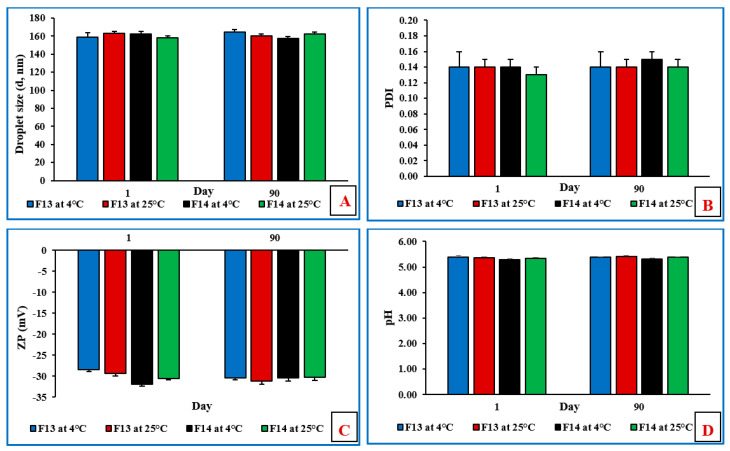
(**A**) Droplet size, (**B**) polydispersity index, (**C**) zeta potential, and (**D**) pH of F13 and F14 formulations over three months of storage at 4 °C and 25 °C (mean ± SD, n = 3).

**Figure 7:**
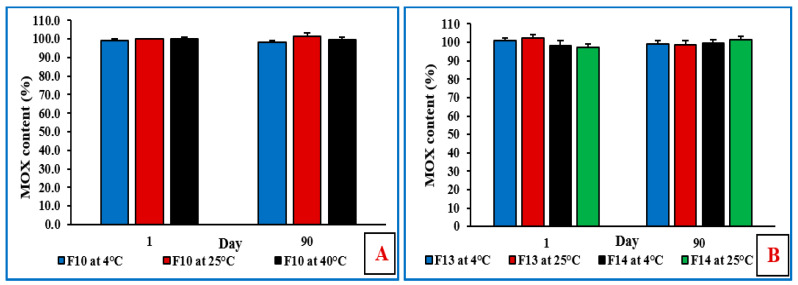
(**A**) Drug content (%) of F10 formulation at 4 °C, 25 °C, and 40 °C; and (**B**) drug content (%) of F13 and F14 formulations at 4 °C and 25 °C over three months of storage (mean ± SD, n = 3).

The physicochemical stability of the non-sterilized MOX-NE and MOX-NEM formulations was evaluated at RF and RT, as well as under accelerated (40 °C) storage conditions for 90 days (last time point tested). The effect of storage conditions on PS, PDI, ZP, pH, and drug content is shown in Figure 5, Figure 6 and Figure 7. None of the three formulations showed any precipitation or cracking upon visual examination. Moreover, there was no significant difference (*p* > 0.05) observed in DS, PDI, pH, ZP, and drug content after three months of storage.

## 4. Conclusions

MOX-loaded NEs and NEM formulations were successfully prepared using HPMC K4M and PVP K29/32 as mucoadhesive agents. MOX-NE and MOX-NEM formulations were sterilized by filtration and moist heat, and the autoclaved formulations were found to be stable for at least one month (last time point tested) after sterilization. Moreover, in vitro release testing showed sustained MOX release profiles from both MOX-NE and MOX-NEM formulations. Ex vivo transcorneal permeation studies revealed a 2.0-fold improvement in permeability and flux from MOX-NE when compared to commercial eyedrops. Both MOX-NEM formulations showed MOX permeability and flux coefficients similar to those of commercial eye drops. Overall, the NE and NEM formulations developed in this investigation appear to be suitable for ocular permeation enhancement of the drug due to the prolonged contact time with the ocular surface and/or extended drug release from the prepared formulation. MOX-NEM formulations could decrease the need for frequent dosing, improve therapeutic outcomes, and increase patient compliance compared to conventional commercial eyedrops. No apparent difference in the functionalities of the two mucoadhesive polymers was observed based on the experiments conducted, although MOX release from the PVP-containing NE formulation was slightly faster than that from the HPMC-containing formulation. Future in vivo studies could elucidate differences in ocular surface retention and delivery based on the mucoadhesive characteristics of the two polymers. In summary, the NE formulations represent a promising MOX delivery platform for the treatment of ocular bacterial infections.

## Figures and Tables

**Table 1:** Oil screening studies for moxifloxacin.

Oil	Solubility	Oil	Solubility
Soybean oil	(−)	Miglyol^®^ 829	(−)
Castor oil	(−)	Labrafac^®^ Lipophile WL 1349	(−)
Oleic acid	(+)	Transcutol^®^ P	(−)
Sesame oil	(−)	Isopropyl Myristate, NF	(−)

(+): MOX is dissolved in the oil and does not precipitate after cooling to RT; (−): MOX either dissolves in the oil but precipitates on cooling to RT or does not dissolve in the oil.

**Table 2:** Composition of different moxifloxacin-loaded nanoemulsions and nanoemulsions with mucoadhesive agent formulations.

Formulation Composition (% *w*/*v*)						
Code *	Oleic Acid	Tween^®^ 80	HPMC K_4_M	PVP K29/32	Milli-Q Water Up to (mL)	Visual Examination
F1	1.0	0.75	–	–	10	Precipitation on day 2
F2	1.0	2.0	–	–	10	Precipitation on day 2
F3	1.0	3.0	–	–	10	Precipitation on day 2
F4	1.0	4.0	–	–	10	Precipitation on day 2
F5	2.5	0.75	–	–	10	Precipitation on day 4
F6	2.5	2.0	–	–	10	Precipitation on day 6
F7	2.5	3.0	–	–	10	Precipitation on day 10
F8	2.5	4.0	–	–	10	Precipitation on day 17
F9	5.0	0.75	–	–	10	Cracking on day 4
F10	5.0	2.0	–	–	10	Stable
F11	5.0	3.0	–	–	10	Stable
F12	5.0	4.0	–	–	10	Stable
F13	5.0	2.0	0.4	–	10	Stable
F14	5.0	2.0	–	0.4	10	Stable

* All formulations contain moxifloxacin (0.5% *w*/*v*) and glycerin (2.25% *w*/*v*).

**Table 3:** Effect of Tween^®^ 80 concentration on droplet size, polydispersity index, zeta potential, and drug content of moxifloxacin-containing NE formulations (mean ± SD, n = 3).

Parameter	F10	F11	F12
Droplet size (d.nm)	174.1 ± 5.9	150.2 ± 2.2	124.7 ± 1.5
Polydispersity index	0.16 ± 0.01	0.14 ± 0.01	0.15 ± 0.02
Zeta potential (mV)	−33.3 ± 0.3	−33.4 ± 0.5	−32.1 ± 1.2
Viscosity (cP)	5.6 ± 0.5	5.6 ± 0.2	5.7 ± 0.4
Drug content (%)	100.8 ± 7.3	101.7 ± 0.3	99.1 ± 3.8

**Table 4:** Droplet size, PDI, zeta potential, drug content, pH, and viscosity of F10, F13, and F14 formulations (mean ± SD, n = 3).

Parameter	F10	F13	F14
Droplet size (d.nm)	165.5 ± 3.2	163.4 ± 2.0	157.8 ± 2.6
Polydispersity index	0.16 ± 0.02	0.14 ± 0.02	0.15 ± 0.01
Zeta potential (mV)	−33.5 ± 1.5	−31.3 ± 0.6	−30.5 ± 0.4
Drug content (%)	99.0 ± 1.2	101.0 ± 1.3	98.3 ± 2.5
pH	5.5 ± 0.1	5.4 ± 0.0	5.4 ± 0.1
Viscosity (cP)	5.6 ± 0.5	26.5 ± 0.9	27.9 ± 0.3

F13 and F14 are NEM formulations with HPMC K4M and PVP K29/30, respectively.

**Table 5:** Effect of autoclaving on the physicochemical characteristics of F10, F13, and F14 formulations before and after sterilization over one month of storage at 4 °C and 25 °C (mean ± SD, n = 3).

Code	Day	Storage at 4 ± 2 °C									
		DS (d, nm)		PDI		ZP (mV)		pH		Drug Content (%)	
		Sterilization Stage									
		Pre	Post	Pre	Post	Pre	Post	Pre	Post	Pre	Post
F10	0	168.3 ± 4.2	162.9 ± 4.8	0.16 ± 0.01	0.16 ± 0.01	−32.5 ± 0.5	−33.5 ± 0.6	5.4 ± 0.1	5.3 ± 0.1	98.3 ± 2.3	101.3 ± 4.3
	30	163.9 ± 3.2	167.5 ± 4.0	0.16 ± 0.02	0.15 ± 0.02	−31.8 ± 1.1	−33.2 ± 0.4	5.4 ± 0.1	5.4 ± 0.1	101.5 ± 3.8	98.2 ± 4.3
F13	0	160.2 ± 3.0	166.2 ± 2.4	0.17 ± 0.01	0.16 ± 0.01	−33.0 ± 0.7	−34.4 ± 0.6	5.3 ± 0.1	5.4 ± 0.1	98.5 ± 4.7	99.1 ± 2.5
	30	165.3 ± 2.3	161.4 ± 3.8	0.16 ± 0.01	0.16 ± 0.03	−32.5 ± 0.3	−34.1 ± 0.2	5.4 ± 0.1	5.4 ± 0.1	102.3 ± 2.6	98.7 ± 2.5
F14	0	172.1 ± 2.8	163.0 ± 3.7	0.17 ± 0.02	0.15 ± 0.02	−33.4 ± 0.6	−31.8 ± 1.0	5.5 ± 0.1	5.3 ± 0.1	99.1 ± 3.0	103.5 ± 3.5
	30	164.7 ± 2.9	167.5 ± 4.2	0.16 ± 0.01	0.17 ± 0.01	−31.7 ± 0.5	−33.1 ± 1.3	5.3 ± 0.1	5.4 ± 0.1	97.5 ± 3.5	98.2 ± 3.0
Code	Day	Storage at 25 ± 2 °C									
F10	0	164.7 ± 1.5	169.9 ± 2.5	0.17 ± 0.01	0.16 ± 0.03	−33.5 ± 1.3	−33.5 ± 2.0	5.5 ± 0.1	5.4 ± 0.1	96.5 ± 4.7	98.8 ± 2.8
	30	160.5 ± 4.6	167.1 ± 2.0	0.17 ± 0.01	0.16 ± 0.01	−31.5 ± 0.3	−31.1 ± 1.2	5.4 ± 0.1	5.4 ± 0.1	98.5 ± 2.5	100.5 ± 1.3
F13	0	166.2 ± 2.4	170.2 ± 5.4	0.16 ± 0.02	0.17 ± 0.01	−32.4 ± 0.5	−31.5 ± 0.7	5.5 ± 0.1	5.4 ± 0.1	102.0 ± 3.5	99.2 ± 2.5
	30	162.5 ± 3.4	166.6 ± 4.2	0.16 ± 0.02	0.15 ± 0.02	−34.7 ± 1.5	−32.0 ± 1.0	5.5 ± 0.1	5.3 ± 0.1	97.6 ± 3.4	99.0 ± 4.7
F14	0	162.0 ± 2.7	168.8 ± 2.4	0.17 ± 0.01	0.15 ± 0.01	−34.4 ± 0.5	−32.7 ± 0.5	5.3 ± 0.1	5.5 ± 0.1	99.0 ± 1.9	98.5 ± 3.1
	30	167.4 ± 4.9	165.4 ± 5.0	0.17 ± 0.01	0.15 ± 0.02	−32.5 ± 0.7	−33.3 ± 0.4	5.4 ± 0.1	5.4 ± 0.1	103.1 ± 2.7	98.9 ± 3.3

**Table 6:** MIC 90 values of lead NE formulations compared to Vigamox^®^ eyedrops and crude drug solution against MRSA.

Formulation	MIC 90 (µg/mL) against MRSA
F10	6.25
Placebo F10	NA
F13	6.25
Placebo F13	NA
F14	6.25
Placebo F14	NA
Vigamox^®^	6.25
MOX-HCL-S	6.25

**Table 7:** Mathematical model fitting of release kinetics of moxifloxacin from F10, F13, and F14 formulations (mean ± SD, n = 3).

Equation	*Q*_0_ − *Q* = *kt*	*ln Q* = *kt*	*Q*_0_ − *Q* = *kt* ^1/2^	*log* (*Q*_0_ − *Q*) = *n log t* + *log k*	
Model	Zero-Order	First-Order	Higuchi	Korsmeyer–Peppas	
	R^2^	R^2^	R^2^	R^2^	n
F10	0.8388	0.9520	0.9786	0.9991	0.7
F13	0.9112	0.9594	0.9906	0.9967	0.6
F14	0.8924	0.9532	0.9885	0.9990	0.6

*Q*_0_ and *Q* represent initial drug content at time *t*_0_ and drug content at time t, respectively; zero-order model: % drug released vs. time; first-order model: amount of drug remaining vs. time; Higuchi model: % drug released vs. square root of time; Korsmeyer–Peppas model: log % drug released vs. log time.

## Data Availability

The data presented in this study are available upon request from the corresponding author.

## References

[1] Teweldemedhin M., Gebreyesus H., Atsbaha A.H., Asgedom S.W., Saravanan M. (2017). Bacterial Profile of Ocular Infections: A Systematic Review. BMC Ophthalmol..

[2] Kowalski R.P., Dhaliwal D.K. (2005). Ocular Bacterial Infections: Current and Future Treatment Options. Expert Rev. Anti Infect. Ther..

[3] Ballouz D., Maganti N., Tuohy M., Errickson J., Woodward M.A. (2019). Medication Burden for Patients with Bacterial Keratitis. Cornea.

[4] Youssef A.A.A., Dudhipala N., Majumdar S. (2022). Dual Drug Loaded Lipid Nanocarrier Formulations for Topical Ocular Applications. Int. J. Nanomed..

[5] Ung L., Bispo P.J.M., Shanbhag S.S., Gilmore M.S., Chodosh J. (2019). The Persistent Dilemma of Microbial Keratitis: Global Burden, Diagnosis, and Antimicrobial Resistance. Surv. Ophthalmol..

[6] Miller D., Cavuoto K.M., Alfonso E.C. (2021). Bacterial Keratitis. Infections of the Cornea and Conjunctiva.

[7] Smith A.F., Waycaster C. (2009). Estimate of the Direct and Indirect Annual Cost of Bacterial Conjunctivitis in the United States. BMC Ophthalmol..

[8] Lemp M.A., Nichols K.K. (2009). Blepharitis in the United States 2009: A Survey-Based Perspective on Prevalence and Treatment. Ocul. Surf..

[9] Durand M.L. (2013). Endophthalmitis. Clin. Microbiol. Infect..

[10] Hooper D.C. (2003). Mechanisms of Quinolone Resistance. Quinolone Antimicrob. Agents.

[11] Kowalski R.P., Dhaliwal D.K., Karenchak L.M., Romanowski E.G., Mah F.S., Ritterband D.C., Gordon Y.J. (2003). Gatifloxacin and Moxifloxacin: An in Vitro Susceptibility Comparison to Levofloxacin, Ciprofloxacin, and Ofloxacin Using Bacterial Keratitis Isolates. Am. J. Ophthalmol..

[12] Belfort R., Gabriel L., Martins Bispo P.J., Muccioli C., Zacharias Serapicos P.C., Clark L., Bell B., Bartell J., Stroman D.W., Höfling-Lima A.L. (2012). Safety and Efficacy of Moxifloxacin-Dexamethasone Eyedrops as Treatment for Bacterial Ocular Infection Associated with Bacterial Blepharitis. Adv. Ther..

[13] Pflugfelder S.C., Karpecki P.M., Perez V.L. (2014). Treatment of Blepharitis: Recent Clinical Trials. Ocul. Surf..

[14] Arshinoff S.A., Modabber M. (2016). Dose and Administration of Intracameral Moxifloxacin for Prophylaxis of Postoperative Endophthalmitis. J. Cataract Refract. Surg..

[15] Vieira I.V., Boianovsky C., Saraiva T.J., Godoy R.B., de Lake J. (2017). Safety and Efficacy of Intracameral Moxifloxacin Injection for Prophylaxis of Endophthalmitis after Phacoemulsification. Arq. Bras. Oftalmol..

[16] Youssef A.A.A., Cai C., Dudhipala N., Majumdar S. (2021). Design of Topical Ocular Ciprofloxacin Nanoemulsion for the Management of Bacterial Keratitis. Pharmaceuticals.

[17] Farkouh A., Frigo P., Czejka M. (2016). Systemic Side Effects of Eye Drops: A Pharmacokinetic Perspective. Clin. Ophthalmol..

[18] Bertino J.S. (2009). Impact of Antibiotic Resistance in the Management of Ocular Infections: The Role of Current and Future Antibiotics. Clin. Ophthalmol..

[19] Pachigolla G., Blomquist P., Cavanagh H.D. (2007). Microbial Keratitis Pathogens and Antibiotic Susceptibilities: A 5-Year Review of Cases at an Urban County Hospital in North Texas. Eye Contact Lens.

[20] Jaiswal M., Dudhe R., Sharma P.K. (2015). Nanoemulsion: An Advanced Mode of Drug Delivery System. 3 Biotech.

[21] Lallemand F., Daull P., Benita S., Buggage R., Garrigue J.-S. (2012). Successfully Improving Ocular Drug Delivery Using the Cationic Nanoemulsion, Novasorb. J. Drug Deliv..

[22] Singh M., Bharadwaj S., Lee K.E., Kang S.G. (2020). Therapeutic Nanoemulsions in Ophthalmic Drug Administration: Concept in Formulations and Characterization Techniques for Ocular Drug Delivery. J. Control. Release.

[23] Ammar H.O., Salama H.A., Ghorab M., Mahmoud A.A. (2009). Nanoemulsion as a Potential Ophthalmic Delivery System for Dorzolamide Hydrochloride. AAPS PharmSciTech.

[24] Sweeney C., Dudhipala N., Thakkar R., Mehraj T., Marathe S., Gul W., ElSohly M.A., Murphy B., Majumdar S. (2021). Effect of Surfactant Concentration and Sterilization Process on Intraocular Pressure–Lowering Activity of Δ9-Tetrahydrocannabinol-Valine-Hemisuccinate (NB1111) Nanoemulsions. Drug Deliv. Transl. Res..

[25] Sweeney C., Dudhipala N., Thakkar R., Mehraj T., Marathe S., Gul W., ElSohly M.A., Murphy B., Majumdar S. (2022). Impact of Mucoadhesive Agent Inclusion on the Intraocular Pressure Lowering Profile of Δ9-Tetrahydrocannabinol-Valine-Hemisuccinate Loaded Nanoemulsions in New Zealand White Rabbits. Int. J. Pharm..

[26] Ludwig A. (2005). The Use of Mucoadhesive Polymers in Ocular Drug Delivery. Adv. Drug Deliv. Rev..

[27] Tundisi L.L., Mostaço G.B., Carricondo P.C., Petri D.F.S. (2021). Hydroxypropyl Methylcellulose: Physicochemical Properties and Ocular Drug Delivery Formulations. Eur. J. Pharm. Sci..

[28] Kurakula M., Rao G.S.N.K. (2020). Pharmaceutical Assessment of Polyvinylpyrrolidone (PVP): As Excipient from Conventional to Controlled Delivery Systems with a Spotlight on COVID-19 Inhibition. J. Drug Deliv. Sci. Technol..

[29] Razzaq S.N., Khan I.U., Mariam I., Razzaq S.S. (2012). Stability Indicating HPLC Method for the Simultaneous Determination of Moxifloxacin and Prednisolone in Pharmaceutical Formulations. Chem. Central J..

[30] Balguri S.P., Adelli G.R., Janga K.Y., Bhagav P., Majumdar S. (2017). Ocular Disposition of Ciprofloxacin from Topical, PEGylated Nanostructured Lipid Carriers: Effect of Molecular Weight and Density of Poly (Ethylene) Glycol. Int. J. Pharm..

[31] Dudhipala N., Ettireddy S., Youssef A.A.A., Puchchakayala G. (2021). Cyclodextrin Complexed Lipid Nanoparticles of Irbesartan for Oral Applications: Design, Development, and In Vitro Characterization. Molecules.

[32] Thakkar R., Komanduri N., Dudhipala N., Tripathi S., Repka M.A., Majumdar S. (2021). Development and Optimization of Hot-Melt Extruded Moxifloxacin Hydrochloride Inserts, for Ocular Applications, Using the Design of Experiments. Int. J. Pharm..

[33] Narang A.S., Delmarre D., Gao D. (2007). Stable Drug Encapsulation in Micelles and Microemulsions. Int. J. Pharm..

[34] Gupta S., Moulik S.P. (2008). Biocompatible Microemulsions and Their Prospective Uses in Drug Delivery. J. Pharm. Sci..

[35] Gao X.-C., Qi H.-P., Bai J.-H., Huang L., Cui H. (2014). Effects of Oleic Acid on the Corneal Permeability of Compounds and Evaluation of Its Ocular Irritation of Rabbit Eyes. Curr. Eye Res..

[36] Sita V.G., Vavia P. (2020). Bromocriptine Nanoemulsion-Loaded Transdermal Gel: Optimization Using Factorial Design, In Vitro and In Vivo Evaluation. AAPS PharmSciTech.

[37] Thakkar H.P., Khunt A., Dhande R.D., Patel A.A. (2015). Formulation and Evaluation of Itraconazole Nanoemulsion for Enhanced Oral Bioavailability. J. Microencapsul..

[38] Youssef A., Dudhipala N., Majumdar S. (2020). Ciprofloxacin Loaded Nanostructured Lipid Carriers Incorporated into In-Situ Gels to Improve Management of Bacterial Endophthalmitis. Pharmaceutics.

[39] Khames A., Khaleel M.A., El-Badawy M.F., El-Nezhawy A.O.H. (2019). Natamycin Solid Lipid Nanoparticles—Sustained Ocular Delivery System of Higher Corneal Penetration against Deep Fungal Keratitis: Preparation and Optimization. Int. J. Nanomed..

[40] Kulkarni S.A., Feng S.-S. (2013). Effects of Particle Size and Surface Modification on Cellular Uptake and Biodistribution of Polymeric Nanoparticles for Drug Delivery. Pharm. Res..

[41] Kou L., Sun J., Zhai Y., He Z. (2013). The Endocytosis and Intracellular Fate of Nanomedicines: Implication for Rational Design. Asian J. Pharm. Sci..

[42] Lin L., Gu Y., Cui H. (2019). Moringa Oil/Chitosan Nanoparticles Embedded Gelatin Nanofibers for Food Packaging against Listeria Monocytogenes and Staphylococcus Aureus on Cheese. Food Packag. Shelf Life.

[43] Makwana S.B., Patel V.A., Parmar S.J. (2016). Development and Characterization of In-Situ Gel for Ophthalmic Formulation Containing Ciprofloxacin Hydrochloride. Results Pharma Sci..

[44] Almáši M., Matiašová A.A., Šuleková M., Beňová E., Ševc J., Váhovská L., Lisnichuk M., Girman V., Zeleňáková A., Hudák A. (2021). In Vivo Study of Light-Driven Naproxen Release from Gated Mesoporous Silica Drug Delivery System. Sci. Rep..

[45] Sterner O., Karageorgaki C., Zürcher M., Zürcher S., Scales C.W., Fadli Z., Spencer N.D., Tosatti S.G. (2017). Reducing Friction in the Eye: A Comparative Study of Lubrication by Surface-Anchored Synthetic and Natural Ocular Mucin Analogues. ACS Appl. Mater. Interfaces.

[46] Abelson M.B., Udell I.J., Weston J.H. (1981). Normal Human Tear PH by Direct Measurement. Arch. Ophthalmol..

[47] Uddin M.S., Mamun A.A., Kabir M.T., Setu J.R., Zaman S., Begum Y., Amran M.S. (2017). Quality Control Tests for Ophthalmic Pharmaceuticals: Pharmacopoeial Standards and Specifications. J. Adv. Med. Pharm. Sci..

[48] Peterson J.M., Fixman M. (1963). Viscosity of Polymer Solutions. J. Chem. Phys..

[49] Astley R., Miller F.C., Mursalin M.H., Coburn P.S., Callegan M.C. (2019). An Eye on Staphylococcus Aureus Toxins: Roles in Ocular Damage and Inflammation. Toxins.

[50] Tayel S.A., El-Nabarawi M.A., Tadros M.I., Abd-Elsalam W.H. (2013). Promising Ion-Sensitive in Situ Ocular Nanoemulsion Gels of Terbinafine Hydrochloride: Design, in Vitro Characterization and in Vivo Estimation of the Ocular Irritation and Drug Pharmacokinetics in the Aqueous Humor of Rabbits. Int. J. Pharm..

[51] Akhter S., Anwar M., Siddiqui M.A., Ahmad I., Ahmad J., Ahmad M.Z., Bhatnagar A., Ahmad F.J. (2016). Improving the Topical Ocular Pharmacokinetics of an Immunosuppressant Agent with Mucoadhesive Nanoemulsions: Formulation Development, in-Vitro and in-Vivo Studies. Colloids Surf. B Biointerfaces.

[52] Khare A., Grover K., Pawar P., Singh I. (2014). Mucoadhesive Polymers for Enhancing Retention in Ocular Drug Delivery: A Critical Review. Rev. Adhes. Adhes..

[53] Agarwal P., Rupenthal I.D. (2016). In Vitro and Ex Vivo Corneal Penetration and Absorption Models. Drug Deliv. Transl. Res..

[54] Shah J., Nair A.B., Jacob S., Patel R.K., Shah H., Shehata T.M., Morsy M.A. (2019). Nanoemulsion Based Vehicle for Effective Ocular Delivery of Moxifloxacin Using Experimental Design and Pharmacokinetic Study in Rabbits. Pharmaceutics.

[55] Gade S., Patel K.K., Gupta C., Anjum M.M., Deepika D., Agrawal A.K., Singh S. (2019). An Ex Vivo Evaluation of Moxifloxacin Nanostructured Lipid Carrier Enriched In Situ Gel for Transcorneal Permeation on Goat Cornea. J. Pharm. Sci..

[56] Sebastián-Morelló M., Calatayud-Pascual M.A., Rodilla V., Balaguer-Fernández C., López-Castellano A. (2018). Ex Vivo Rabbit Cornea Diffusion Studies with a Soluble Insert of Moxifloxacin. Drug Deliv. Transl. Res..

